# The ribonucleotidyl transferase USIP-1 acts with SART3 to promote U6 snRNA recycling

**DOI:** 10.1093/nar/gkv196

**Published:** 2015-03-09

**Authors:** Stefan Rüegger, Takashi S. Miki, Daniel Hess, Helge Großhans

**Affiliations:** 1 Friedrich Miescher Institute for Biomedical Research, Maulbeerstrasse 66, CH-4058 Basel, Switzerland; 2 University of Basel, Petersplatz 1, CH-4003 Basel, Switzerland

## Abstract

The spliceosome is a large molecular machine that serves to remove the intervening sequences that are present in most eukaryotic pre-mRNAs. At its core are five small nuclear ribonucleoprotein complexes, the U1, U2, U4, U5 and U6 snRNPs, which undergo dynamic rearrangements during splicing. Their reutilization for subsequent rounds of splicing requires reversion to their original configurations, but little is known about this process. Here, we show that ZK863.4/USIP-1 (U Six snRNA-Interacting Protein-1) is a ribonucleotidyl transferase that promotes accumulation of the *Caenorhabditis elegans* U6 snRNA. Endogenous USIP-1–U6 snRNA complexes lack the Lsm proteins that constitute the protein core of the U6 snRNP, but contain the U6 snRNP recycling factor SART3/B0035.12. Furthermore, co-immunoprecipitation experiments suggest that SART3 but not USIP-1 occurs also in a separate complex containing both the U4 and U6 snRNPs. Based on this evidence, genetic interaction between *usip-1* and *sart-3*, and the apparent dissociation of Lsm proteins from the U6 snRNA during spliceosome activation, we propose that USIP-1 functions upstream of SART3 to promote U6 snRNA recycling.

## INTRODUCTION

A set of five U snRNAs, U1, U2, U4, U5 and U6 snRNAs, is central to most pre-mRNA splicing events in eukaryotes (1). These RNAs occur in ribonucleoprotein (RNP) complexes that comprise a core set of seven Sm proteins in the case of U1, U2, U4 and U5, and seven Sm-like (Lsm) proteins in the case of U6 (2). The splicing process involves extensive changes of U snRNP structure and composition (1,3). For example, extensive base-pairing with U4 maintains U6 in an inactive conformation during its recruitment as a U4/U6.U5 tri-snRNP to the pre-spliceosome, which contains pre-mRNA bound to U1 and U2 snRNPs. Major rearrangements of U6 then lead to disruption of the U4–U6 snRNA base-pairing in favor of U6–U2 snRNA base-pairing, resulting in release of U4 snRNA (3). Moreover, U6 binding to the 5′ splice site displaces the U1 snRNA leading to its release from the spliceosome (3).

Following execution of the splicing step, U2, U5 and U6 snRNPs and the resected intron lariat are released and further disassembled through mechanisms that are not well understood (1). Reuse of the snRNPs for further rounds of splicing thus requires regeneration of their distinct, initial conformations and interactions. For the U6 snRNP, this ‘recycling’ includes the reformation of a U4/U6 snRNP. Genetic studies in yeast implicated the RNA-binding protein Prp24p in this process (4,5) and subsequent studies have shown that Prp24p promotes annealing of the U4 and U6 snRNAs by structurally rearranging the U6 snRNA (6,7). For the related human protein, SART3/p110/TIP110, a similar function as a U4/U6 snRNP annealing factor has been described (8,9). In addition, however, SART3 (squamous-cell carcinoma antigen recognized by T cells-3 (10)) has been implicated in transcriptional activation (11) and repression (12), deubiquitination (13,14), and small RNA silencing pathways (15).

U6 stands out among U snRNAs, and non-coding RNAs more generally, by virtue of its high degree of conservation (16), exhibiting 75% sequence identity between yeast and human and 91% between *Caenorhabditis elegans* and human. This may reflect its central role in the splicing process where it is thought to contribute, together with the U2 snRNA, to the active site of the spliceosome (17). U6 snRNA is also unique among U snRNAs in that it is transcribed by RNA polymerase III (Pol III) rather than Pol II (18). Because Pol III transcription is terminated by a stretch of four to six consecutive deoxythimidines (19), the 3′ end of U6 thus consists of a short stretch of uridines. For mammalian U6, the precise number of Us appears to be variable, ranging from 0 to 12 U residues (20–22), possibly reflecting the antagonistic activities of exonucleolytic shortening and non-templated extension.

The uridine tail at the 3′ end of the nascent Pol III transcript is bound by the La protein, which protects it from exonucleolytic degradation (23). In the case of U6 snRNA, the U-tail is subsequently bound by Lsm proteins in both yeast and mammals, replacing the La protein (24–26). Lsm proteins in turn stabilize U6 snRNA (27) and enhance the binding of Prp24p/SART3 to U6 snRNA to facilitate U4/U6 snRNA annealing (25,28,29). Hence, the uridine tail at the 3′ end of U6 snRNA is pivotal for its stability and recycling.

Here, we report the role of a previously uncharacterized protein, ZK863.4/USIP-1, in U6 snRNA metabolism. USIP-1 is a ribonucleotidyl transferase that binds U6 snRNA and promotes its accumulation. In addition, USIP-1 interacts genetically and physically with SART3, but, unlike SART3, does not bind to the U4/U6 di-snRNP. Hence, these data suggest that USIP-1 functions to promote U6 snRNA recycling with, and upstream of, SART3.

## MATERIALS AND METHODS

### Strains

Strains were cultured using standard methods on OP50-seeded NGM plates (30). The Bristol N2 strain was used as wild-type. Mutant and transgenic strains generated for this study are listed in Supplementary Table S3. Most lines were obtained by Mos1-mediated Single-Copy transgene Insertion as previously described (MosSCI; (31,32)). All strains have been backcrossed two times, unless indicated otherwise. *usip-1(tm1897)* animals were obtained from Dr Shohei Mitani and backcrossed two times. The resulting strain was called HW1251. Strains HW1340 and HW1342 contain extrachromosomal arrays of GFP/3xFLAG-tagged fosmids which were obtained from (33).

### Cloning

The *sart-3* and *usip-1* gene were amplified from genomic *C. elegans* DNA by PfuUltra II Fusion HS DNA Polymerase (Agilent Technologies, Santa Clara, CA, USA) according to the supplier's protocol using specific primers (Supplementary Table S4). Point mutations D183A and D185A were introduced to *usip-1* (amplified from genomic DNA) by site-directed mutagenesis using PfuUltra II Fusion HS DNA Polymerase according to (34) with specific primers (Supplementary Table S4). D183A and D185A were introduced to the cDNA of *usip-1* for recombinant protein expression using Gibson Assembly according to (35) with specific primers (Supplementary Table S4).

### RNAi

The RNAi clone against *sart-3* was obtained from (36). RNAi was carried out by feeding worms with HT115 bacteria expressing dsRNA of *sart-3* or an insertless plasmid (L4440) as negative control according to (37).

### Single-copy transgene insertion

DNA fragments were inserted into pCFJ210 (for chromosome I), pCFJ150 (for chromosome II) or pCFJ201 (for chromosome IV) vectors by Multisite Gateway Technology (Life Technologies, Carlsbad, CA, USA) according to the supplier's protocol and as detailed in Supplementary Table S3. *Mos1*-mediated Single-Copy transgene Insertion (MosSCI) was performed according to previous reports (31,32). Successful insertion of transgenes was verified by polymerase chain reaction (PCR).

### Fluorescence-based worm sorting

As a consequence of the lethal phenotype of worms homozygous for the *xe3* allele (*xe3*/*xe3*), these worms were maintained as heterozygotes (*xe3*/+) utilizing the *nT1[qls51]* balancer containing a fluorescent marker (pharyngeal GFP) (strain HW1337; Supplementary Table S3). This allows differentiation between *xe3*/*xe3* worms, which lack pharyngeal GFP, and *xe3*/+ and +/+ worms, which have pharyngeal GFP. Homogenous populations of *xe3*/*xe3* worms were obtained by sorting out GFP-containing worms from a mixed population on a COPAS BIOSORT device (Union Biometrica, Holliston, MA, USA).

### Antibodies and western blotting

Polyclonal, affinity purified anti-SART3 was generated by SDIX (Newark, DE, USA) using DNA immunization of rabbits against a polypeptide (amino acids 1–163). Antibodies were used at the following dilutions: rabbit anti-*C. elegans* SART3 (Q5635) 1:2000, mouse anti-Actin (clone C4, MAB1501, Millipore, Billerica, MA, USA) 1:10 000, mouse anti-GFP (mixture of clones 7.1 and 13.1, Roche, Penzberg, Germany) 1:2000, mouse anti-FLAG (clone M2, F1804, Sigma–Aldrich, St Louis, MO, USA) 1:2000. Western blotting was performed as previously described (38). Band intensities were quantified using ImageJ software (NIH, Bethesda, MD, USA).

### RNA isolation and northern blotting

Worms were mixed with TRI Reagent (Molecular Research Center, Cincinnati, OH, USA) and freeze–thawed as described previously (39). The RNA was extracted according to the manufacturer's instructions. Total RNA or RNA from IP's (see ‘Immunoprecipitation’ section) was separated on a 8 M urea–10% polyacrylamide gel electrophoresis (PAGE) and transferred to a Hybond-NX membrane (GE Healthcare, Little Chalfont, UK) by semi-dry blotting. Cross-linking was carried out by UV irradiation using a UV Stratalinker 1800 (Stratagene, La Jolla, CA, USA) followed by baking (1 h at 80°C). Single-stranded DNA probes were designed with Unique Probe Selector (http://array.iis.sinica.edu.tw/ups/index.php). Sequences of probes are given in Supplementary Table S4. Probes were 5′ end-labeled with ATP-γ-[32P] and polynucleotide kinase according to standard protocols. Hybridization was carried out overnight in 4× SSPE (0.6 M NaCl, 40 mM NaH_2_PO_4_, 4 mM ethylenediaminetetraacetic acid (EDTA)), 7% sodium dodecyl sulfate (SDS), in the presence of 0% to 32% formamide at 37°C.

### Immunoprecipitation

Mixed stage worms (HW1339 or HW1342, see Supplementary Table S3) were lysed with a Dounce Tissue Grinder (BC Scientific, Miami, FL, USA) in 30 mM HEPES/KOH pH 7.4, 100 mM KCl, 1.5 mM MgCl_2_, 0.1% Triton X-100 and protease inhibitors (Protease Inhibitor Cocktail Tablets, EDTA-free, Roche). Lysates were cleared at 16 000 x *g* for 15 min. RNase A-treated samples were additionally incubated with 0.1 mg/ml RNase A (Sigma–Aldrich) for 1 h at 4°C. For anti-FLAG IP, 2 mg lysate was incubated with anti-FLAG M2 magnetic beads (Sigma–Aldrich) for 2 h. Washes were performed in lysis buffer. Elution was achieved by incubation with 1 mg/ml FLAG peptide (Sigma–Aldrich). For RNA extractions, TRI Reagent (Molecular Research Center) was directly added to the magnetics beads. For anti-SART3 IP, lysates were incubated with 5 μg purified antibody (anti-SART3) for 1 h. Protein A sepharose beads (Roche) were added for 2 h. Washes were performed in lysis buffer. Complexes were eluted by heating the beads in sample loading buffer containing reducing agent for 10 min at 70°C.

### Mass spectrometry

TCA precipitated and acetone washed protein pellets were dissolved in 0.5 M Tris–HCl pH 8.6, 6 M guanidinium hydrochloride, reduced in 16 mM tris(2-carboxyethyl)phosphine (TCEP) for 30 min, and alkylated in 35 mM iodoacetamide for 30 min in the dark. The proteins were digested at 37°C with trypsin (Promega, Madison, USA) after 6× dilution in 50 mM Tris–HCl pH 7.4, 5 mM CaCl_2_ overnight. The resulting peptides were separated on a 75 μm × 10 cm Magic C18 column (Michrom, Bioresources, Auburn, USA) with an Agilent 1100 Nanoflow LC System (Agilent, Palo Alto, CA, USA). The LC was connected to a LTQ Orbitrap Velos (Thermo Scientific). Mascot (Matrix Science, London, UK) searching UniProt data base version 2012_09 was used to identify the peptides.

### Microscopy

DIC and fluorescent images were obtained using an Axio Observer Z1 microscope and AxioVision SE64 (release 4.8) software (Carl Zeiss, Oberkochen, Germany). Stereoscopic images were obtained by a M205 A stereo microscope (Leica, Solms, Germany).

### MosDEL

The *xe3* allele was obtained by following the protocol established by (40). A targeting plasmid was created using the Multisite Gateway Technology (Life Technologies). Specific primers (Supplementary Table S4) were used to amplify the left (2039 bp) and right (2988 bp) homology regions from genomic DNA and amplicons were cloned into pDONRP4-P1R and pDONRP2R-P3, respectively. Together with pENTR221 containing and *unc-119* rescue gene a pDESTR4-R3 targeting plasmid was created. The targeting plasmid was injected at 50 ng/ul into strain HW1350, a *Mos1*-engineered strain (IE5820 containing the ttTi5820 allele) obtained from the NemaGENETAG consortium (41) which we crossed into an *unc-119(ed3)* mutant background. Following injection, wild-type moving worms were screened for successful integration of the transgene by PCR (Supplementary Table S4 for primer sequences). The isolated deletion allele was balanced with *nT1[qls51]* and the resulting strain was called HW1337 (Supplementary Table S3).

### Recombinant protein expression and purification

cDNA for ZK863.4/USIP-1, either wild-type or containing the point mutations D183A and D185A (USIP-1^cd^), was cloned into pOPINE (introducing a C-terminal hexa-histidine tag). USIP-1/USIP-1^cd^ was transformed into *Escherichia coli* BL21 cells. A starter culture (GS96 medium supplemented with 0.05% glycerol, 1% glucose, 50 μg/ml carbenicillin) was inoculated and incubated for 11 h at 37°C and 225 rpm. An expression culture (ZYP-5052 medium for auto-induction) was inoculated with the starter culture (1:100) and incubated for 4 h at 37°C followed by 20 h at 20°C. Cells were harvested by spinning for 30 min at 6500 x *g* at 4°C. The pellet was resuspended in wash buffer (20 mM NaH_2_PO_4_, 500 mM NaCl, 20 mM imidazole, pH 7.5) containing 0.5% Tween (v/w). The suspension was sonicated in the presence of Benzonase (3 U/ml culture) and protease inhibitors (Protease Inhibitor Cocktail Tablets, EDTA-free, Roche). The lysate was cleared by spinning for 30 min at 30 000 x *g* at 4°C. USIP-1/USIP-1^cd^ was purified from the cleared lysate using a HisTrap HP column (5 ml) (GE Healthcare) on an ÄKTApurifier 10 HPLC (GE Healthcare). Recombinant protein was recovered from the column with elution buffer (20 mM NaH_2_PO_4_, 500 mM NaCl, 500 mM imidazole, pH 7.5), concentrated with Amicon Ultra-4 centrifugal filter units (10 000 K, Millipore) and stored at −20°C in 10 mM Tris–HCl pH 7.5, 100 mM NaCl, 1 mM Dithiothreitol (DTT), 50% glycerol.

### Transferase assay

Twenty micrograms recombinant USIP1 or USIP-1^cd^ were added to 200 ng of a synthetic RNA 22mer (AGCCGCAUUUCGUAGUGAUAUU) in the presence of 1 mM UTP (or, when appropriate, ATP, CTP or GTP), RNasin (40 U/μl) (Promega, Madison, USA), and reaction buffer (10 mM Tris–HCl pH 7.5, 50 mM NaCl, 10 mM MgCl_2_, 1 mM DTT) in a total volume of 15 μl. Samples were incubated at 25°C for 0–10 min and the reaction was stopped by adding 950 μl TRI Reagent (Molecular Research Center). The RNA was extracted according to the manufacturer's instructions and the reaction products were separated by 8 M urea–15% PAGE. The RNA was visualized by incubating with SYBR Gold. In Figure 4D, 30 ng gel-purified U6 RNA was incubated with recombinant protein in the presence of 1.6 μM UTP-α-[32P] (800 Ci/mmol) mixed with 200 μM cold UTP for 5 min at 25°C. The reaction products were separated by 8 M urea–10% PAGE and visualized by autoradiography. *In vitro* transcribed U6 was obtained using the MEGAscript Kit (Life Technologies) on synthetic double-stranded DNA corresponding to the U6 sequence with five deoxythymidines at the 3′ end and including the T7 promoter at the 5′ end. Note that the T7 promoter introduces three artificial guanosine residues at the 5′ end during transcription.

**Figure 1.**
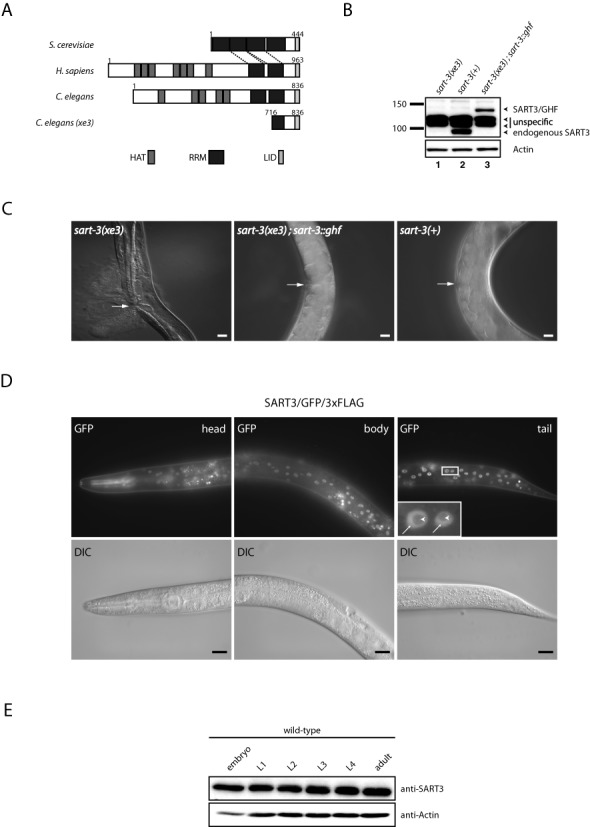
*B0035.12* encodes an essential, nuclear protein orthologous to SART3. (**A**) Schematic representation of the domain structure of Prp24p from *Saccharomyces cerevisiae*, SART3 from *Homo sapiens*, and SART3 from *Caenorhabditis elegans*. *xe3* denotes an N-terminal truncation allele of SART3. Numbers indicate amino acid positions. HAT = half a TPR, RRM = RNA recognition motif, LID = Lsm interaction domain. (**B**) Western blot of lysates extracted from L4 stage worms. Transgenic and endogenous SART3 were detected with an affinity-purified polyclonal antibody against SART3 in lysates of worms homozygous for the *xe3* allele (lane 1), which we obtained by sorting, wild-type worms (lane 2), and *xe3* homozygous mutant worms rescued by transgenic *sart-3* containing a C-terminal GFP/His/FLAG-tag (GHF) (lane 3). (**C**) Differential interference contrast (DIC) micrographs. Adult worms homozygous for the *xe3* allele burst through the vulva (left panel). By contrast, *xe3* worms expressing transgenic *sart-3::gfp::his::flag* (middle panel), like wild-type worms (right panel), do not burst. Arrows point to the vulva. Scale bar, 20 μm. (**D**) Fluorescence and DIC micrographs of L4 stage worms expressing C-terminally GFP/3xFLAG-tagged SART3 from a fosmid. Pharynx signal in the head (top left panel) arises from an RFP co-injection marker leaking into the GFP channel. Arrows point to nucleoplasm, arrow heads point to nucleolus. Scale bar, 20 μm. (**E**) Western blot with lysates from wild-type worms extracted at different time points during development. L1 = larval stage 1, etc.

**Figure 2.**
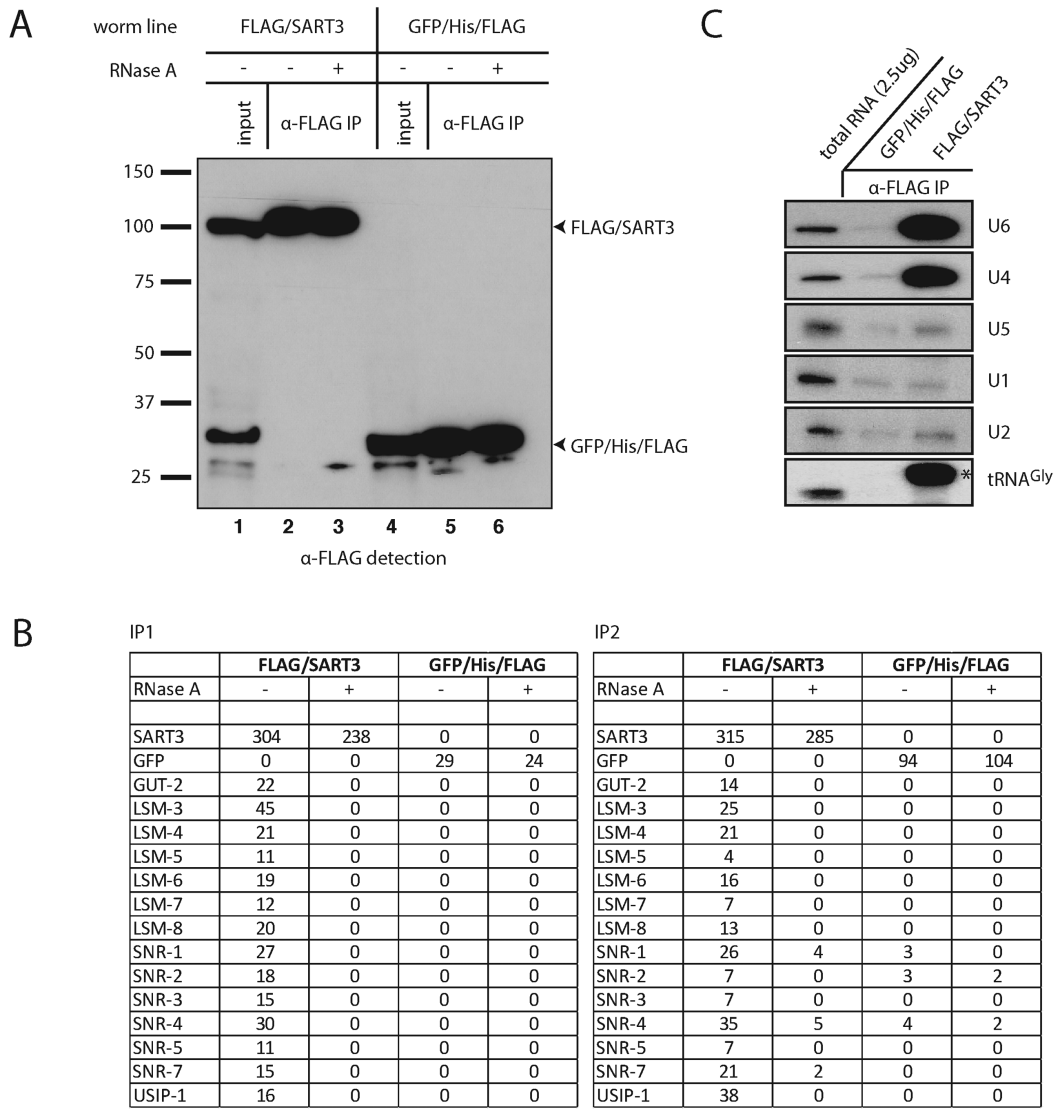
SART3 co-immunoprecipitates U4 and U6 snRNPs. (**A**) Western blot of anti-FLAG co-immunoprecipitation (co-IP) of N-terminally FLAG-tagged SART3 (lanes 1–3) and a GFP/His/FLAG control construct (lanes 4–6). Seven percent of input and 15% of IPs were loaded on a gel. (**B**) Mass spectrometry results of FLAG/SART3 co-IPs. Numbers of spectra mapping uniquely to a given protein are indicated. IP1: protein extraction using dounce homogenizer, RNase A treatment performed at room temperature, eluate concentration by TCA precipitation. IP2: protein extraction using mortar and pestle, RNase A treatment performed at 4°C, eluate concentration by speed vac. GUT-2 is homologous to human Lsm2 and the SNR proteins to Sm proteins. The percentage coverage for these proteins is provided in Supplementary Table S1. (**C**) Northern blot of RNA extracted from eluates obtained by an anti-FLAG co-IP on lysates from worms expressing the indicated transgene. Two milligram total protein was used as input for the co-IP and 100% of the co-immunoprecipitated RNA was loaded on the gel. 2.5 μg of total RNA were loaded as a reference. Subsets of probes were applied to the membrane to detect different RNAs simultaneously; the asterisk indicates an unspecific band detected with the probe against U6.

**Figure 3.**
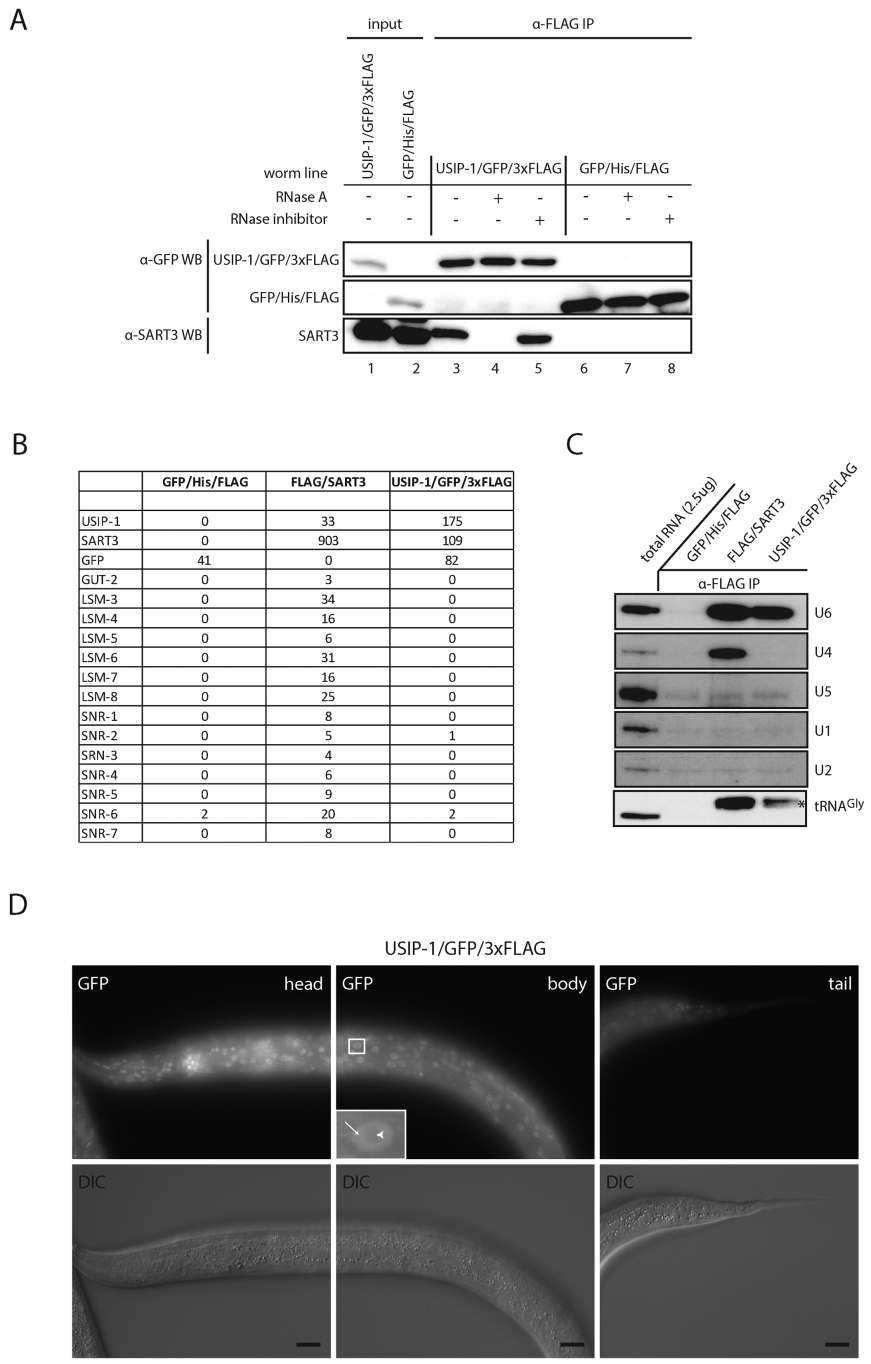
SART3 and USIP1/ZK863.4 are in a U6 snRNA-containing complex. (**A**) Western blot of anti-FLAG co-IP of C-terminally GFP/3xFLAG-tagged USIP-1 (lanes 1 and 3–5) and a C-terminally His/FLAG-tagged GFP control construct (lanes 2 and 6–8). USIP-1/GFP/3xFLAG and GFP/His/FLAG constructs were detected with anti-GFP. Endogenous SART3 was detected with a polyclonal antibody against SART3. Two percent of input and 70% of IPs were loaded on a gel. (**B**) Proteins identified by mass spectrometry following IP of FLAG-tagged SART3, USIP-1 or GFP. The latter serves as a negative control (see additional proteins in Supplementary Table S2). Numbers of spectra mapping uniquely to a given protein are indicated. (**C**) Northern blot of RNA extracted from eluates obtained by an anti-FLAG co-IP on lysates from worms expressing the indicated transgene. Two milligram total protein was used as input for the co-IP and 100% of the co-immunoprecipitated RNA was loaded on the gel. 2.5 μg of total RNA were loaded as a reference. Subsets of probes were applied to the membrane to detect different RNAs simultaneously; the asterisk indicates an unspecific band detected with the probe against U6. (**D**) Fluorescence and DIC microscopy of L4 stage worms expressing C-terminally GFP/3xFLAG-tagged USIP-1 from a fosmid. Arrows point to nucleoplasm, arrow heads point to nucleolus. Scale bar, 20 μm.

**Figure 4.**
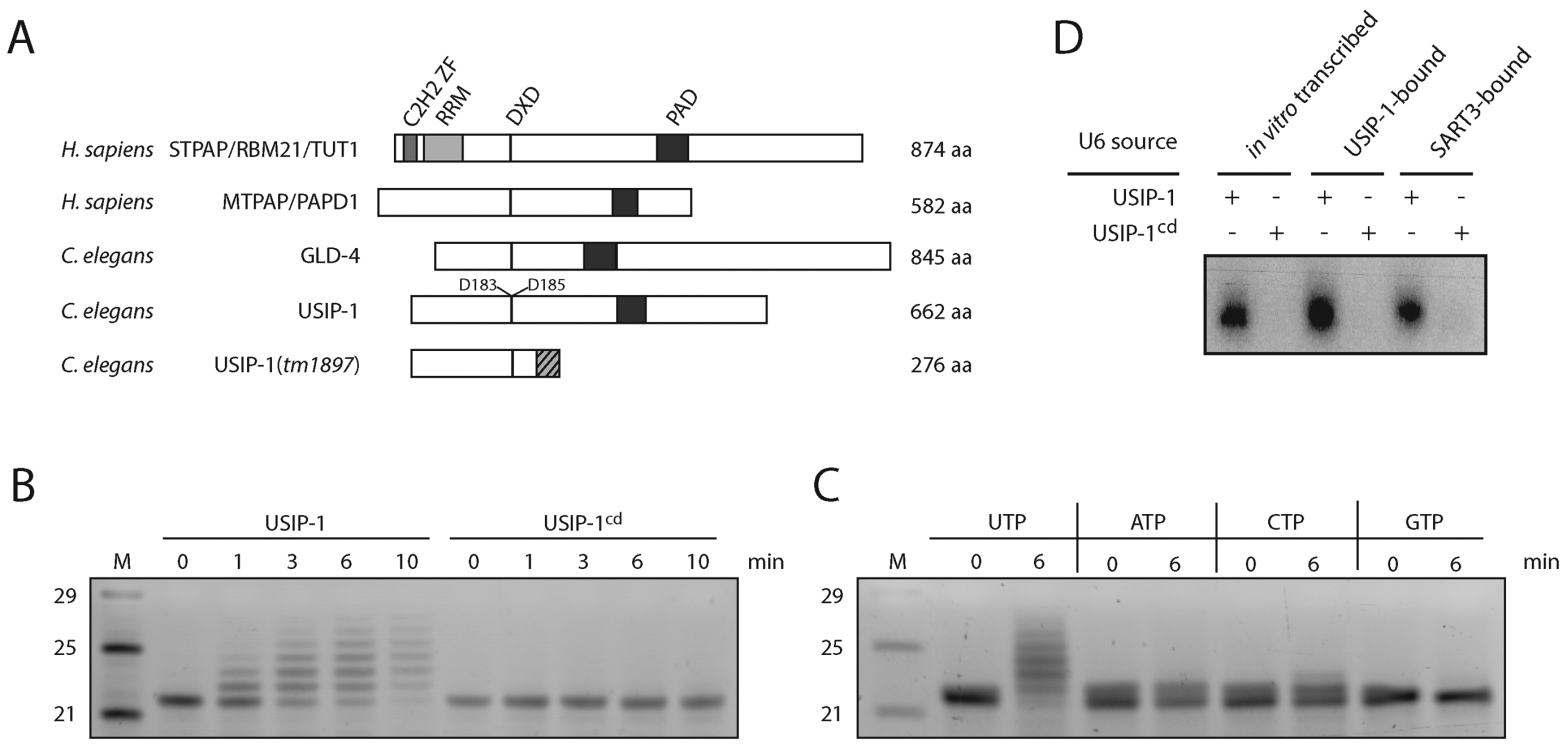
USIP-1 is a terminal uridylyl transferase. (**A**) Schematic representation of the domain structure of STPAP/RBM21/TUT1 (UniProt: STPAP_HUMAN) and MTPAP/PAPD1 (PAPD1_HUMAN) from *H. sapiens*, and the *C. elegans* proteins GLD-4 (GLD4_CAEEL) and USIP-1 (Q23652_CAEEL). *tm1897* denotes a deletion allele of USIP-1 that leads to a frame shift (striated) and premature termination codon at position 276. C_2_H_2_ ZF = C_2_H_2_-type (classical) zinc finger (ZF), RRM = RNA recognition motif, PAD = PAP-associated domain, DXD: Amino acid motif required for catalytic activity where D = aspartic acid and X = any amino acid. (**B**) Assay to test terminal transferase activity of recombinant USIP-1 and USIP-1^cd^. Twenty micrograms of recombinant protein was added to 200 ng of a synthetic 22-nucleotide-long RNA substrate in the presence of 1 mM UTP. The reaction was stopped by addition of Trizol between 0 and 10 min. Shown is a SYBR Gold staining of a 15% urea–polyacrylamide gel (inverted picture). M = Marker. (**C**) Transferase assay similar to (B) with different nucleotide triphosphates. (**D**) Autoradiography of transferase activity of recombinant USIP-1 or USIP-1^cd^ on gel-purified U6 RNA from the indicated sources using radioactively labeled UTP-α-^[32]^P.

### 3′ RACE

To determine the 3′-terminal sequence of U6 snRNA, we ligated the 3′ RNA adapter from the TruSeq Small RNA Sample Preparation Kit (Illumina, San Diego, CA, USA) onto 1 μg total RNA according to the supplier's protocol. Ligated RNA was reverse transcribed for 30 min at 42°C followed by 1 h at 50°C using a primer that introduces a primer binding site for subsequent amplification (Supplementary Table S4) and components of the TruSeq Small RNA Sample Preparation Kit (Illumina).The reverse transcriptase was inactivated by incubation of the sample at 70°C for 15 min. The cDNA was diluted 1:10 and U6 was amplified using a U6-specific primer and a primer complementary to the region introduced by reverse transcription (Supplementary Table S4) by Taq DNA polymerase (New England Biolabs, Ipswich, MA, USA). PCR amplicons were cloned into the pCR4TOPO vector according to the supplier's protocol (Life Technologies) and sequenced.

## RESULTS

### *sart-3* is a ubiquitously and constitutively expressed gene required for *C. elegans* viability

Although U6 snRNP recycling is arguably the best understood function of SART3, this protein has also been implicated in other pathways, including, by virtue of its interaction with the human AGO1 and AGO2 proteins, in small RNA silencing (15). To gain further insight into the physiological functions of SART3, we studied the *C. elegans* protein B0035.12. Although the overall sequence identity with the human protein is low (26%), the domain composition is highly conserved (Figure 1A) and includes several HAT (half a TPR) repeats in the N-terminal part, two RNA recognition motifs (RRMs) in the C-terminal part, and an Lsm interaction domain (LID) at the C-terminus. For this and other reasons that we will describe below, we named the *B0035.12* gene *sart-3*, encoding the SART3 protein.

We used the MosDEL technique for targeted gene disruption (40) to delete amino acids 1–715 of the endogenous *sart-3* locus, generating the *sart-3(xe3)* mutant allele (Figure 1A). An affinity-purified polyclonal antibody against SART3 failed to detect a band for full-length SART3 in a Western blot with lysates from L4 stage worms homozygous for the *xe3* allele (Figure 1B, compare lanes 1 and 2), which we obtained through fluorescence-based sorting of the progeny of *sart-3(xe3)*/*nT1[qls51]* animals carrying a GFP-marked balancer (see ‘Materials and Methods’ section for details). As the antibody was generated against an N-terminal polypeptide (amino acids (aa) 1–166), the accumulation of a C-terminal fragment (aa 716–836) remains hypothetically possible, but seems unlikely given the lack of an ATG start codon in the corresponding DNA sequence.

Homozygously *sart-3(xe3)* mutant animals died as young adults from vulval rupturing with a penetrance of 100% (*n* = 20) (Figure 1C, left panel). Expression of a single-copy integrated transgene encoding SART3 C-terminally fused to a GFP/His/FLAG triple tag (GHF) and expressed from a ubiquitous *dpy-30* promoter (P*dpy-30::sart-3::ghf*) restored the wild-type situation (Figure 1C, middle and right panels). Western blot analysis verified that the levels of the SART3/GHF and endogenous SART3 are comparable (Figure 1B, compare lanes 2 and 3). Hence, we conclude that lethality is a consequence of *sart-3* mutation, revealing that *sart-3* is essential for *C. elegans* development and viability.

Visual inspection of *dpy-30::sart-3::ghf* animals indicated nuclear steady-state localization of the tagged protein. However, it was possible that intracellular localization was influenced by the use of the heterologous *dpy-30* promoter, which we had used because *sart-3* is the second gene in a three-gene operon (Wormbase WS244; (42)). Therefore, to confirm this localization and examine spatial and temporal expression patterns of *sart-3*, we generated a distinct transgenic strain that produced SART3/GFP/3xFLAG from a fosmid (33), and thus under endogenous *cis* regulatory control (Figure 1D). Whereas mammalian SART3 accumulates strongly in nuclear foci termed Cajal/Coiled Bodies (CBs) (43,44), we observed diffuse nucleoplasmic staining for *C. elegans* SART3 (Figure 1D). This finding is consistent with the fact that the *C. elegans* genome lacks an obvious orthologue of coilin (Wormbase WS244; (42)), a key structural component of CBs (45).

The transgene also revealed widespread, presumably ubiquitous, and constitutive accumulation of SART3. To confirm the latter, we examined endogenous SART3 levels at different time points during development by western blot, which revealed sustained *sart-3* expression (Figure 1E). Taken together, SART3 is a constitutively and ubiquitously expressed nuclear protein that is essential for *C. elegans* vulval integrity and thus viability.

### *C. elegans* SART3 associates with the U4/U6 snRNP

Although vulval bursting may occur through dysregulation of diverse pathways, it is also a hallmark of miRNA dysfunction in *C. elegans* (46,47), possibly supporting a notion of SART3 functioning in this pathway (15). Whereas nuclear steady-state localization argued against a function in cytoplasmic Argonaute complexes, a role in miRNA biogenesis remained possible. Hence, to understand the key pathways of SART3 function in *C. elegans*, we precipitated functional, N-terminally FLAG-tagged SART3 (FLAG/SART3) or as a negative control a GFP/His/FLAG triple tag from mixed stage worm lysates using an anti-FLAG antibody (Figure 2A). Precipitates were eluted by incubation with FLAG peptide and examined by mass spectrometry. We were unable to identify interaction with any known miRNA pathway components. By contrast, we detected a full complement of Lsm2 (GUT-2 in *C. elegans*) through Lsm8 proteins (Figure 2B and Supplementary Table S1), which make up the protein core of the U6 snRNP (2). We did not observe Lsm1, which forms a related complex on mRNAs destined for degradation but absent from the U6 snRNP (2). To confirm that SART3 bound to the entire U6 snRNP, we performed northern blot analysis of RNA co-immunoprecipitated with this protein, and observed a strong enrichment of U6 snRNA relative to tRNA^Gly^ (Figure 2C).

In addition to Lsm proteins, FLAG/SART3 also immunoprecipitated the Sm proteins that form the protein core of the U1, U2, U4 and U5 snRNAs, with only SNR-6/Sm E being undetectable (Figure 2B). We thus tested whether FLAG/SART3 also interacted with additional U snRNAs. We found that SART3 did not appreciably bind U1, U2 or U5 snRNAs (Figure 2C). However, it efficiently co-immunoprecipitated U4 snRNA (Figure 2C), suggesting that the binding to the Sm proteins could be mediated through U4. Consistent with this notion, RNase A treatment of lysates prevented co-immunoprecipitation of both Lsm and Sm proteins with FLAG/SART3 (Figure 2B). Collectively, these data are consistent with the notion that *C. elegans* SART3, like its mammalian and yeast counterparts, functions in recycling of post-splicing U6 snRNP by reannealing U4 and U6 snRNAs to generate a di-snRNP.

### ZK863.4/USIP-1 is in a complex with SART3 and U6 snRNA

In addition to Lsm and Sm proteins, FLAG/SART3 reproducibly co-immunoprecipitated a novel protein, ZK863.4/USIP-1 (Figure 2A). We verified that, reciprocally, ZK863.4/USIP-1 tagged with GFP/3xFLAG (33), expressed from a multicopy fosmid transgene array, was capable of co-immunoprecipitating SART3 (Figure 3A and B). As the interaction of ZK863.4/USIP-1 and SART3 was sensitive to RNase A treatment (Figure 3A), we wondered if it was mediated by U6 and/or U4 snRNAs. Indeed, analysis of co-precipitating RNA by Northern blotting revealed the presence of U6 snRNA (Figure 3C), leading us to term this protein USIP-1 for U-Six snRNA Interacting Protein. However, U4 snRNA was not co-precipitated (Figure 3C), and Sm proteins were not enriched above the background of a GFP/His/Flag control immunoprecipitation (Figure 3B). Hence, unlike SART3, USIP-1 does not appear to occur on the U4/U6 di-snRNP. In fact, as Lsm proteins did not detectably co-immunoprecipitate with USIP-1 either, it appears that USIP-1 binds preferentially to U6 snRNA that is not assembled into the canonical U6 snRNP. Additional proteins co-immunoprecipitated with SART3 and USIP-1 but are not further pursued in this study (Supplementary Table S2). Similar to SART3, we found the USIP-1/GFP/3xFLAG multicopy array to be expressed diffusely throughout the nucleoplasm but depleted from the nucleolus and expressed constitutively and ubiquitously across different tissues (Figure 3D).

### USIP-1 is a terminal nucleotidyl transferase

Sequence analysis of USIP-1 suggested that it was a ribonucleotidyl transferase, as it contained both the DXD motif and PAD domain characteristic of this class of enzymes (48) (Figure 4A). To determine whether USIP-1 was a functional enzyme, we produced recombinant wild-type protein through expression in *E. coli* (Supplementary Figure S1). Additionally, we produced a variant containing a double D->A mutation in the DXD motif, USIP-1 (D183A, D185A), which is expected to abrogate Mg^2+^ binding and thus enzymatic activity (48,49). We will refer to the catalytic-dead protein as USIP-1^cd^ in the following. Incubation of the wild-type enzyme with a synthetic RNA oligonucleotide revealed distributive ribonucleotidyl transferase activity as shown by extension of the substrate (Figure 4B). This activity depended on the recombinant wild-type protein as USIP-1^cd^ was inactive (Figure 4B). Moreover, examination of the activity with different nucleotide triphosphates revealed activity preferentially in the presence of UTP (Figure 4C). In conclusion, USIP-1 is a *bona fide* terminal uridylyl transferase (TUTase).

### USIP-1 can extend endogenous U6 snRNA

The 3′ end of mature U6 snRNA is usually chemically modified in a manner that renders it resistant to periodate-induced shortening through beta-elimination (50). In *C. elegans*, a vast majority of U6 snRNA molecules is ‘blocked’ at its 3′ end in this manner, but the identity of the relevant modification is not known (50). To examine whether endogenous U6 snRNA was a suitable substrate of USIP-1, we gel-purified this RNA from USIP-1 and SART3 co-immunoprecipitates, respectively. When equimolar amounts of USIP-1- or SART3-bound U6 snRNA, or *in vitro*-transcribed U6 snRNA were incubated with USIP-1 and radiolabeled α-^[32]^P-UTP, labeling occurred on all three RNAs and was similar in extent (Figure 4D). By contrast, no labeling occurred when USIP-1^cd^ was used. We conclude that USIP-1 is capable of extending endogenous U6 snRNA. Moreover, 3′-monophosphorylated synthetic RNA was refractory to tailing by USIP-1 (Supplementary Figure S3), consistent with the finding that the U6 snRNA of *C. elegans*, unlike that of other organisms, does not carry a 2′,3′-cyclo- or 3′-monophosphate blocking group (50). Interestingly, another common RNA modification, 2′-*O*-methylation, similarly abrogated USIP-1-mediated tailing (Supplementary Figure S3), suggesting the presence of a more unusual modification on the U6 snRNA 3′ end, which remains to be identified.

### SART3 depletion is synthetically lethal with loss of USIP-1 TUTase activity

Genetic modulation, i.e. enhancement or suppression of mutant phenotypes, is an established way to identify genes functioning in shared or parallel pathways. Hence, we sought to use modulation of *usip-1* and *sart-3* mutant phenotypes to confirm that both these genes function in U6 snRNA pathways. In order to look for genetic interaction, we made use of a partial deletion mutant of *usip-1, usip-1(tm1897)*, kindly provided by Dr Shohei Mitani. The *tm1897* allele is a deletion of 542 bases that leads to a frame shift at amino acid position 233 (I233T) and to a premature termination codon shortly after (S276Stop), suggesting it to be a hypomorph or null mutation (Figure 4A).

**Figure 5.**
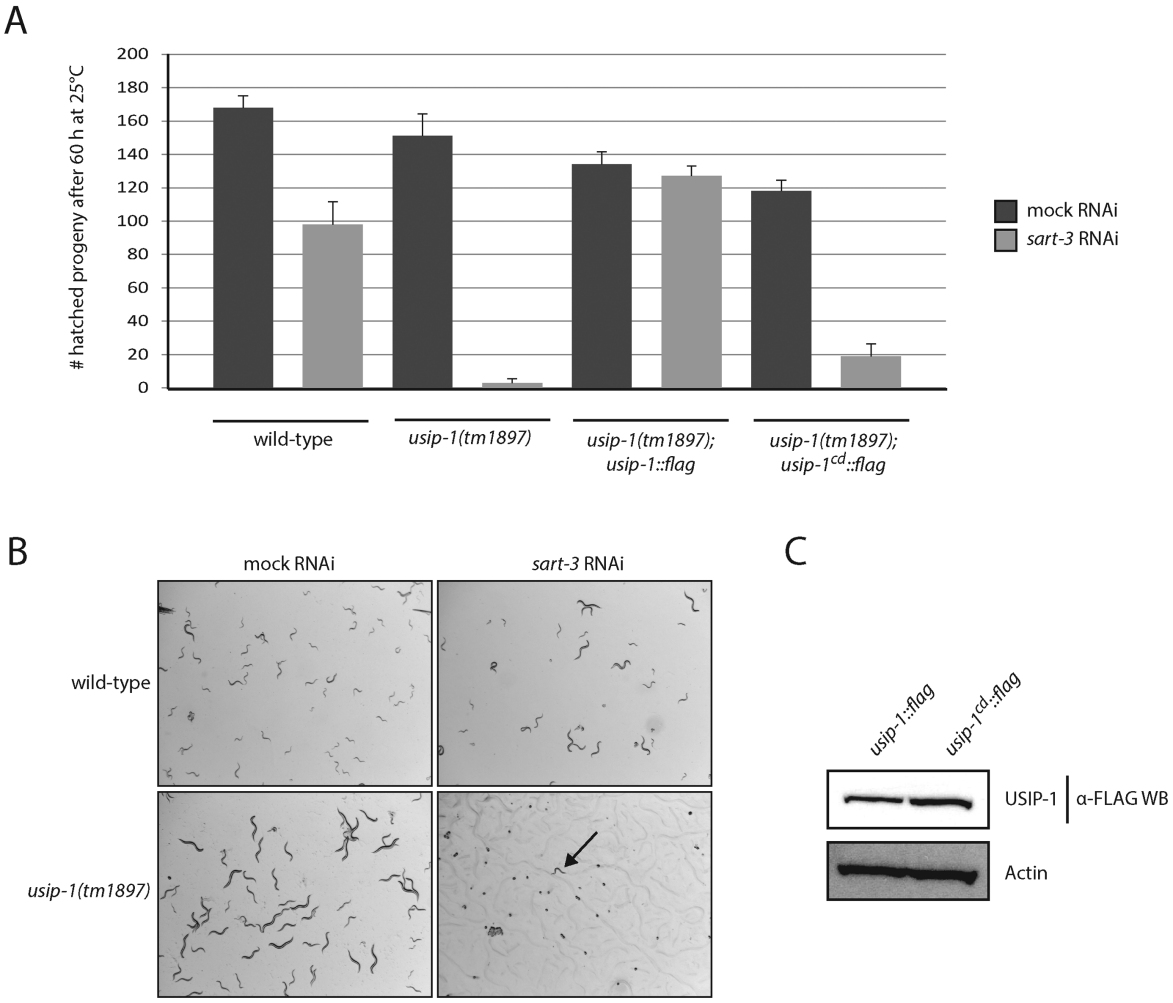
Synthetic embryonic lethality occurs when simultaneously compromising SART3 and USIP-1 activity. (**A**) Wild-type worms or worms homozygous for the *tm1897* allele were exposed to mock or *sart-3* RNAi at the L1 stage (P0 generation) and cultured at 25°C. After 60 h, the hatched progeny (F1 generation) was counted. *n* = 3, error bars indicate SEM. (**B**) Wild-type worms or worms homozygous for the *tm1897* allele were exposed to mock or *sart-3* RNAi at the L1 stage (P0 generation) and cultured at 25°C. Pictures of the next generation (F1 generation) were taken after 96 h revealing hatched F1's (that were arrested due to lack of food though) for wild-type; mock RNAi, wild-type; *sart-3* RNAi and *usip-1(tm1897);* mock RNAi but arrested embryos or L1 stage worms (arrow) for *usip-1(tm1897); sart-3* RNAi. (**C**) Western blotting shows similar protein levels for transgenic, FLAG-tagged wild-type USIP-1 or mutant USIP-1^cd^. Both transgenes are expressed in a *usip-1(tm1897)* background and were detected by an anti-FLAG antibody.

*usip-1(tm1897)* mutant animals were viable and overtly fine. Extensive depletion of SART3 by RNAi (Supplementary Figure S2), unlike its complete deletion, failed to yield overt phenotypes beyond a moderately reduced brood size when scored after 60 h at 25°C (Figure 5A). However, when *usip-1(tm1897)* mutant animals were additionally treated with *sart-3(RNAi)*, embryonic lethality ensued (Figure 5A and B). In order to exclude the possibility that the unhatched embryos were just developmentally delayed and would hatch at a later time point, we assessed them after 96 h at 25°C and found them still arrested (Figure 5B). Closer inspection revealed that the embryos arrested at various developmental stages (data not shown). The few worms that did hatch under *usip-1(tm1897)*; *sart-3(RNAi)* conditions arrested at the L1 stage (Figure 5B). Hence, these data are consistent with the notion that USIP-1, like SART3, is important for U6 snRNA function.

The genetic interaction permitted us to test the relevance of the TUTase activity of USIP-1 for its *in vivo* function. To do so, we expressed transgenes that encoded wild-type USIP-1 or USIP-1^cd^, respectively, in *usip-1(tm1897)* mutant animals, which we then exposed to RNAi against *sart-3*. Strikingly, although USIP-1 and USIP-1^cd^ accumulated to similar levels (Figure 5C), only the wild-type protein supported viability (Figure 5A). Hence, TUTase activity of USIP-1 appears essential when SART3 activity is decreased.

### Loss of USIP-1 leads to U6 snRNA destabilization

**Figure 6.**
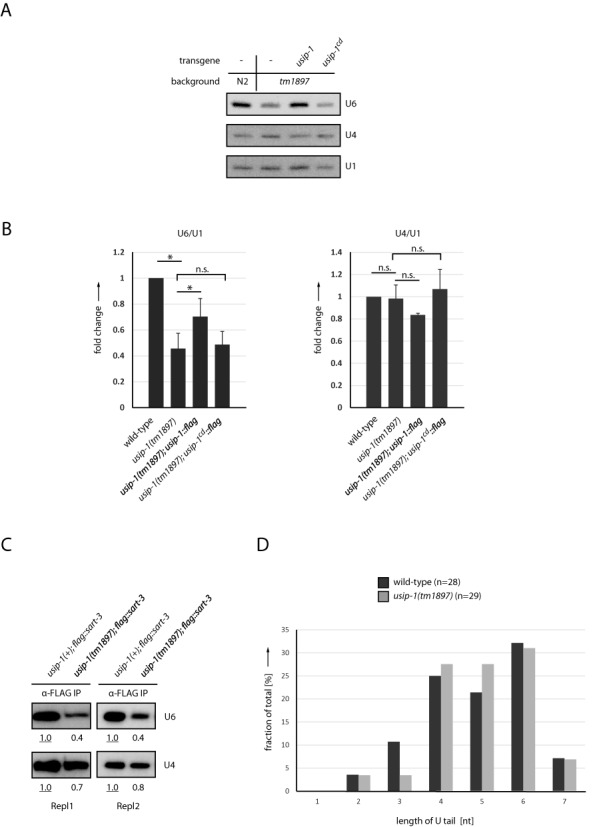
USIP-1 stabilizes U6 snRNA. (**A**) Northern blot with RNA extracted from wild-type worms (N2) or *usip-1(tm1897)* worms. The latter contain either no transgene or transgenic *usip-1* or *usip-1^cd^*. (**B**) Quantification of northern blots as shown in (A) from experiments with four biological replicates, mean + SEM. *P-value < 0.05 by a paired two-tailed *t*-test; n.s., not statistically significant. (**C**) Northern blot with RNA isolated from immunoprecipitated FLAG/SART3. FLAG/SART3 was immunoprecipitated from wild-type worms (*usip-1(+)*) or *usip-1(tm1897)* worms. Two replicates are shown. Quantification of bands is relative to the underlined value, which has been set to 1. (**D**) 3′ end sequences of U6 snRNA from wild-type and *usip-1(tm1897)* worms were determined by 3′ RACE analysis and grouped as indicated according to length of the oligo-U tail. [Note that only tail sequences consisting entirely of Us were used for the analysis depicted here, but that comparable results were obtained when including the occasional tail sequences that contain As in addition to Us (data not shown).]

With genetic and biochemical evidence thus strongly implicating USIP-1 in U6 snRNA function, we sought to determine whether USIP-1 affected U6 snRNA levels. Using Northern blotting, we observed decreased U6 snRNA levels in *usip-1(tm1897)* mutant relative to wild-type animals (Figure 6A and Supplementary Figure S4). The defect in U6 snRNA accumulation was partially rescued by transgene-directed expression of USIP-1 but not USIP-1^cd^ (Figure 6A and B). Similar findings were made when U6 snRNA bound to SART3 was examined (Figure 6C). Moreover, although U4 snRNA levels in total lysates remained unaffected by loss of USIP-1 or its catalytic activity, its abundance in SART3 co-IPs was reproducibly reduced when USIP-1 was absent (Figure 6C). We conclude that USIP-1 and its TUTase activity are required for U6 snRNA accumulation and that defective U6 snRNA accumulation may be a cause of the synthetic lethality that occurs in *usip-1(tm1897)*; *sart-3(RNAi)* animals.

Although no obvious differences in U6 snRNA migration occurred in RNA from *usip-1(tm1897)* relative to wild-type animals (Figure 6A), minor differences in the oligo-U tail length of this RNA might not be detected at the resolution of our gel system. Hence, we performed rapid amplification of cDNA ends (RACE) analysis and examined U6 snRNA 3′ ends by sequencing. In wild-type animals, most molecules contained an oligo-U tail of four to six nucleotides; with few examples of shorter tails (Figure 6D). This is consistent with a stretch of four to six T's serving as the termination sequence of RNA polymerase III, and the preferential binding of Lsm proteins to U_4_ tails. Surprisingly, this pattern was unaltered in *usip-1* mutants (Figure 6D). This finding suggests that U6 snRNA molecules that cannot be extended to their normal length of ≥4 U nucleotides at the 3′ end due to loss of USIP-1 are rapidly degraded.

## DISCUSSION

**Figure 7.**
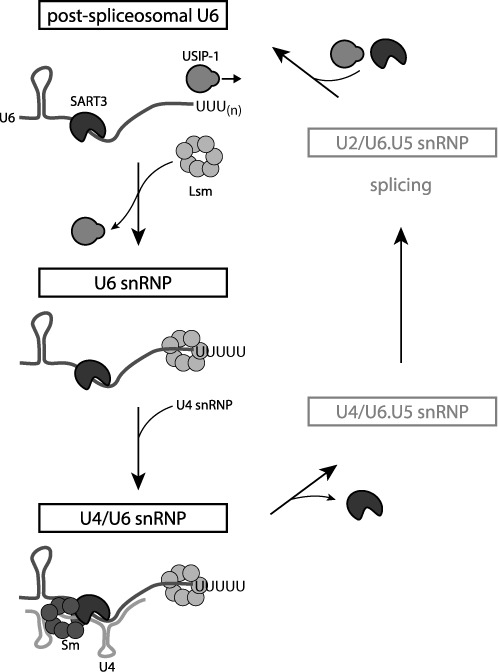
Model of the life cycle of U6 snRNA. Additional snRNP-associated proteins (1) are omitted for the sake of simplicity. See text for details.

Whereas the splicing process and mechanisms of spliceosome activation are understood in substantial detail, much less is known about processes that promote reutilization of the spliceosome for additional rounds of splicing (1). We propose here that USIP-1 is a novel U6 snRNA recycling factor. We note that at this point we cannot formally exclude a role of USIP-1 in U6 snRNA biogenesis. However, the fact that USIP-1 interacts both physically and genetically with a *bona fide* U6 snRNA recycling factor strongly implies that U6 snRNA recycling is a major function of USIP-1. Indeed, when integrating the results that we present in the current study with previous data on U4/U6 di-snRNP regeneration (5,8,9,51), a U6 snRNA recycling pathway begins to emerge (Figure 7). In particular, we propose that USIP-1 binds to ‘post-spliceosomal’ U6 snRNA, devoid of Lsm proteins. Subsequently or coincidentally, SART3 is recruited to this complex. The complex then matures to acquire Lsm proteins as well as the U4 snRNP (24,27,29), associated with Sm proteins, while ejecting USIP-1.

This model is supported as follows: *In vitro* studies previously suggested the possibility that Lsm proteins dissociate from the U6 snRNP during spliceosome activitation (52,53), thus generating the presumed USIP-1 substrate. Moreover, because Lsm proteins bind to the very 3′ terminus of U6 snRNA (26) to which a TUTase also needs to bind to extend the RNA, we can anticipate that binding of TUTase and Lsm proteins is mutually incompatible. Finally, SART3 occurs in two distinct complexes that contain either only U6 snRNA or both U4 and U6 snRNA (8), presumably corresponding to early and late steps of U6 snRNA recycling.

It was previously suggested that Lsm proteins facilitated recruitment of SART3 to the U6 snRNA because *in vitro* their strong binding to this snRNA stabilized the weak binding of SART3 to U6 (28). However, these experimental data are equally consistent with a model where binding of SART3 to U6 can initiate in the absence of Lsm proteins, possibly facilitated by USIP-1 binding to the U6 snRNA, but then gaining stability, and possibly specificity (7), through interaction with the Lsm toroid. At any rate, the presence of both SART3 and Lsm proteins may then maintain the U6 snRNA, now devoid of USIP-1, in a configuration that promotes annealing to the U4 snRNA (24,27,29).

The destabilization of U6 snRNA upon loss of USIP-1 might be explained by the fact that binding of the Lsm complex to U6 snRNA requires the latter's 3′-terminal oligo-U tail (24–26). Failure to reconstitute the U6 snRNA oligo-U tail to its normal length of four to five nucleotides will thus impair binding of the Lsm complex. This, in turn, might make the U6 snRNA more susceptible to nucleolytic decay, in particular through an exonucleolytic attack on its now freely accessible 3′ end. Although not proof, the fact that trimmed U6 snRNA molecules fail to accumulate in *usip-1* mutant animals is indeed consistent with their being an unstable RNA species.

Although no direct one-to-one orthologue of USIP-1 can be identified in mammalian cells, it appears most similar to mitochondrial poly(A) RNA polymerase (MTPAP/PAPD1) (54) and speckle targeted PIP5K1A-regulated poly(A) polymerase (STPAP/RBM21/TUT1) (55) (Figure 4A). Interestingly, STPAP was originally identified by virtue of its biochemical activity, the specific addition of uridine nucleotides to U6 snRNA, and named U6 TUTase (56,57). However, its physiological function remained unknown, and was further obscured by subsequent work suggesting that a select set of mRNAs, not U6 snRNA, were its substrates, which it preferentially extended with adenosine, not uridine (55). Although we do not understand the basis of these inconsistent findings, we note that siRNA-mediated depletion of STPAP was recently shown to decrease the length heterogeneity of cellular U6 snRNA in HeLa cells (58) suggesting that U6 snRNA might indeed be one of its cellular substrates. Hence, we speculate that STPAP/U6 TUTase function analogously to USIP-1. The divergence in sequence, functions, and domain organization between USIP-1 and STPAP may then reflect peculiarities in U6 snRNA 3′ end formation pathways among eukaryotes (50,59).

## SUPPLEMENTARY DATA

Supplementary Data are available at NAR Online.

SUPPLEMENTARY DATA
