# Supplementary material for: The ribonucleotidyl transferase USIP-1 acts with SART3 to promote U6 snRNA recycling
Source: Nucleic Acids Res. 2015 Mar 9;43(6):3344–57. doi: 10.1093/nar/gkv196 (PMC4381082; doi:10.1093/nar/gkv196)
Supplement: SUPPLEMENTARY DATA [file supp_43_6_3344__index.html]

The ribonucleotidyl transferase USIP-1 acts with SART3 to promote U6 snRNA recycling — The ribonucleotidyl transferase USIP-1 acts with SART3 to promote U6 snRNA recycling — SUPPLEMENTARY DATA 

# The ribonucleotidyl transferase USIP-1 acts with SART3 to promote U6 snRNA recycling

## SUPPLEMENTARY DATA

**Files in this Data Supplement:**

- SUPPLEMENTARY DATA
