# Supplementary material for: The ribonucleotidyl transferase USIP-1 acts with SART3 to promote U6 snRNA recycling
Source: Nucleic Acids Res. 2015 Mar 9;43(6):3344–57. doi: 10.1093/nar/gkv196 (PMC4381082; doi:10.1093/nar/gkv196)
Supplement: SUPPLEMENTARY DATA [file supp_gkv196_nar-02990-a-2014-File009.pdf]

SUPPLEMENTARY FIGURES AND TABLES

S. Rüegger et al. The Ribonucleotidyl Transferase USIP-1 Acts with SART3 to Promote U6 snRNA Recycling. Nucleic Acids Res (2015)

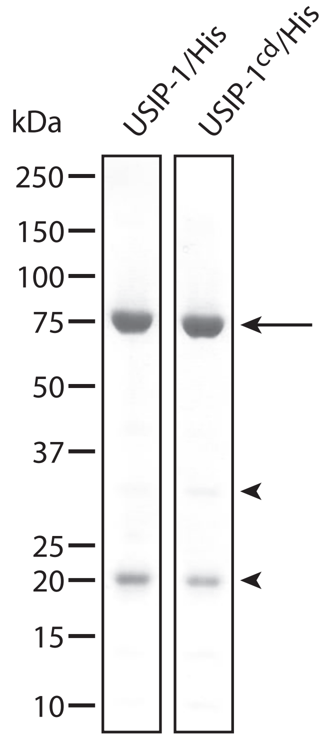

Figure S1. Purity of recombinant USIP-1.
Coomassie staining of recombinant USIP-1 and USIP-1cd purified through a C-terminal His-Tag using a HisTrap column. An arrow points to USIP-1, which has a calculated mass of 75 kDa; arrowheads point to residual contaminations.

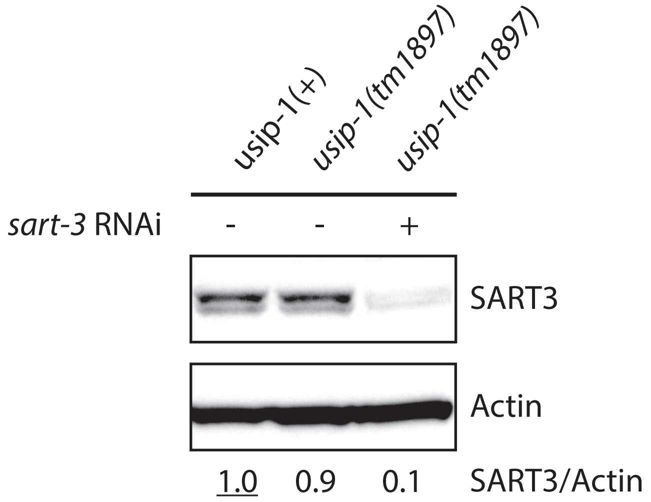

Figure S2. Knock-down efficiency of *sart-3*.
Western blot of lysates obtained from wild-type worms (*usip-1(+)*) exposed to mock RNAi (lane 1), *usip-1(tm1897)* worms exposed to mock RNAi (lane 2), and *usip-1(tm1897)* worms exposed to *sart-3* RNAi (lane 3). Quantification of band signals is relative to the underlined value, which has been set to 1.

Figure S3

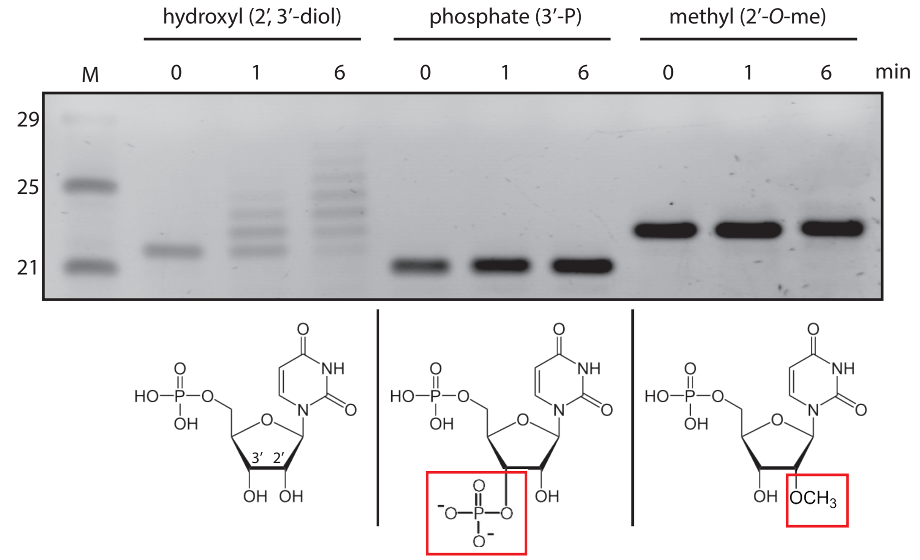

Figure S3. Activity of USIP-1 on different RNA 3’ ends.
Transferase assay (see methods) adding recombinant USIP-1 to a synthetic 22-nucleotide-long RNA substrate containing a uridine at the 3’ end that is either unmodified (2’,3’-diol), 3’-monophosphorylated (3’-P), or 2’-*O*-methylated (2’-*O*-me). Note that the 2’-*O*-methylated substrate is 23 (not 22) nucleotides long containing an additional uridine at the 3’ end. Shown is a SYBR Gold staining of a 15% urea-polyacrylamide gel (inverted picture). M = Marker

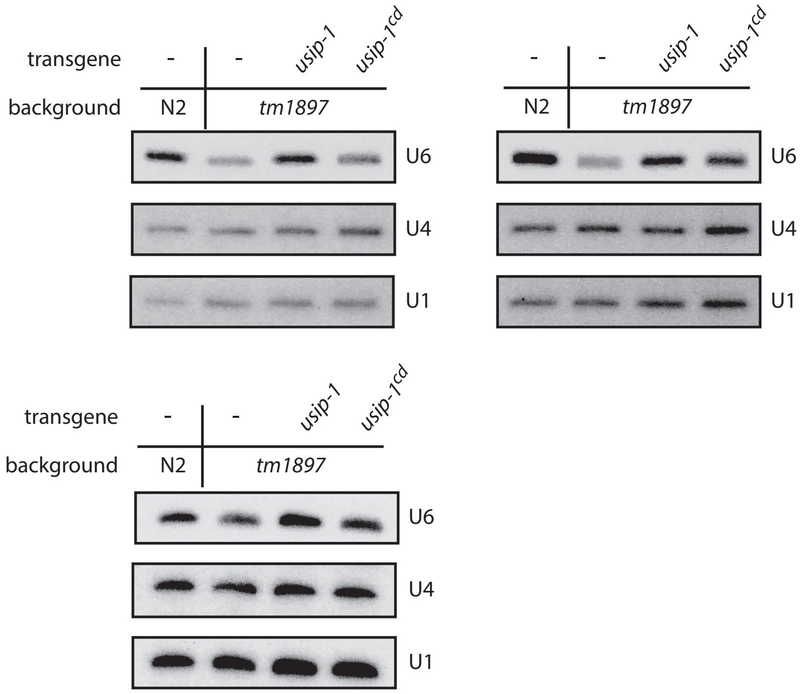

Figure S4. Replicate 2-4 of Northern blot shown in figure 6A.

Table S1. Coverage in percent of protein sequence of indicated proteins in IP1 and IP2 shown in figure 2B.

IP1

	FLAG/SART3		GFP/His/FLAG	
RNase A	no	yes	no	yes
SART3	57	50	0	0
GFP	0	0	58	46
GUT-2	80	0	0	0
LSM-3	75	0	0	0
LSM-4	28	0	0	0
LSM-5	87	0	0	0
LSM-6	36	0	0	0
LSM-7	33	0	0	0
LSM-8	84	0	0	0
SNR-1	50	0	0	0
SNR-2	33	0	0	0
SNR-3	25	0	0	0
SNR-4	59	0	0	0
SNR-5	42	0	0	0
SNR-7	61	0	0	0
USIP-1	19	0	0	0

IP2

	FLAG/SART3		GFP/His/FLAG	
RNase A	no	yes	no	yes
SART3	53	53	0	0
GFP	0	0	78	75
GUT-2	60	0	0	0
LSM-3	71	0	0	0
LSM-4	41	0	0	0
LSM-5	21	0	0	0
LSM-6	60	0	0	0
LSM-7	19	0	0	0
LSM-8	72	0	0	0
SNR-1	51	0	0	0
SNR-2	0	0	0	0
SNR-3	42	0	0	0
SNR-4	56	0	0	26
SNR-5	48	0	0	0
SNR-7	53	40	0	0
USIP-1	33	0	0	0

	IP1		IP2	
	LSM	SNR	LSM	SNR
average % coverage	60.4	45	49.1	41.7

Table S2. Additional proteins bound to USIP-1 and SART3.

Additional proteins identified by mass spectrometry in the co-IP shown in figure 3B enriched by ≥ 10-fold in either the USIP-1 or SART3 IP over the GFP/His/FLAG negative control. Table is ranked according to the USIP-1/GFP ratio. The numbers in the table indicate the quantity of spectra mapping uniquely to a given protein.

C. elegans	*H. sapiens*	*S. cerevisiae*	GFP/His/FLAG	USIP-1/GFP/3xFLAG	FLAG/SART3
swsn-1	SMARCC1	SWI3	0	28	8
atp-2	ATP5B	ATP2	0	25	8
pde-6	PDE8A	PDE2	0	24	6
hsp-6	HSPA9	SSC1	0	21	9
rpl-34	RPL34	RPL34B	0	18	11
rpn-2	PSMD1	RPN2	0	18	5
rpl-9	RPL9	RPL9B	0	17	11
rpn-3	PSMD3	RPN3	0	16	9
sip-1	CRYAB		0	16	0
rpl-26	RPL26	RPL26B	0	14	11
rpl-19	RPL19	RPL19A	0	14	5
rpl-32	RPL32	RPL32	0	13	9
atp-4	ATP5J		0	12	8
cct-5	CCT5	CCT5	0	12	5
copb-2	COPB2	SEC27	0	12	0
eef-2	EEF2	EFT1	0	11	6
nap-1	NAP1L1	NAP1	0	11	5
dlst-1	DLST	KGD2	0	11	5
rpn-8	PSMD7	RPN8	0	11	5
F18C12.3	SRFBP1	USO1	0	11	4
cpsf-1	CPSF1	CFT1	0	11	2
copa-1	COPA	COP1	0	11	0
rpl-28	RPL28		0	10	8
pas-4	PSMA7	PRE6	0	10	5
cgh-1	DDX6	DHH1	0	10	3
cey-1	YBX3	NOP1	0	6	10

Table S3. Worm strains

strain name	genotype	comment
**gfp control**		
HW781	EG5003, xeSi17[Pxrn-2::gfp::his::flag::xrn-2 3', unc-119(+)] IV	
**sart-3 lines***		
HW1008	EG6701, xeSi55[Pdpy-30::sart-3::gfp::his::flag::xrn-2 3', unc-119(+)] I	
HW1337	sart-3(xe3)/nT1[qls51] IV	
HW1338	HW1008; sart-3(xe3) IV	
HW1339	EG6699, xeSi126[Pdpy-30::flag::sart-3::gpd-operon::gfp::his-58::tbb-2 3', unc-119(+)] II	
HW1340	EG6699, xeEx386(WRM0622D_C09::gfp::3xflag; Pmyo-2::mCherry)	gfp::3xflag tagged B0035.12 (sart-3) on fosmid
HW1341	HW1339; sart-3(xe3)/nT1[qls51] IV	
HW1350	unc-119(ed3) III; ttTi5820 IV	
**usip-1 lines***		
HW1251	ZK863.4(tm1897) V	
HW1342	EG6699, xeEx387(WRM0610A_C05::gfp::3xflag)	gfp::3xflag tagged ZK863.4 (usip-1) on fosmid
HW1343	EG6699, xeSi127[Pdpy-30::ZK863.4::flag::gpd-operon::gfp::his-58::tbb-2 3'), unc-119(+)] II	
HW1344	EG6699, xeSi128[Pdpy-30::ZK863.4(D183A/D185A)::flag::gpd-operon::gfp::his-58::tbb-2 3'), unc-119(+)] II	
HW1345	HW1343; ZK863.4(tm1897) V	
HW1346	HW1344; ZK863.4(tm1897) V	

Table S4. Oligonucleotide primer & northern probe sequences

Lower case letters = gateway recombination sites

**Cloning**	**Forward (5'->3')**	**Reverse (5'->3')**
sart-3 (genomic DNA)	ggggacaagtttgtacaaaaaagcaggcttgATGTCCGATGTGGATATGG	ggggaccactttgtacaagaaagctgggtgATTTTTCATAAACATTTTACGG
flag::sart-3 (genomic DNA)	ggggacaagtttgtacaaaaaagcaggctgcATGGATTATAAAGATGATGATGACAAGTCCGATGTGGATATG	ggggaccactttgtacaagaaagctgggtgTTAATTTTTCATAAACATTTTAC
usip-1 (genomic DNA)	ggggacaagtttgtacaaaaaagcaggcttgATGTCTTCAAACTTGCAACTGG	ggggaccactttgtacaagaaagctgggtgTTATGGCCAAGTTGGGGCTGC
**Genotyping**		
tm1897	CGCCTCCGTGCGCACTTGAG	GTTATGCTGTGAAAACAAGC
**Site-directed mutagenesis**		
usip-1(D183A) (on genomic DNA)	GGCGAAATGGTTACTCAGCGATTGTAAGTTGTTAG	CTAACAACTTACAATCGCTGAGTAACCATTTCGCC
usip-1(D185A) (on genomic DNA)	CAATAATGTTTTTAGGCGATTAACGTGGAATCAG	CTGATTCCACGTTAATCGCCTAAAAACATTATTG
usip-1 amplicon 1 (on cDNA, Gibson cloning)	GGCTTCTGGCGTGTGACCGGCGGCTCTAGAGCCTCTGCTAACC	CTGATTCCACGTTAATCGCAATCGCTGAGTAACCATTTCG
usip-1 amplicon 2 (on cDNA, Gibson cloning)	CGAAATGGTTACTCAGCGATTGCGATTAACGTGGAATCAG	CGAAATATTATGAGTAATATCCATGGGATCCATCAACATCAACGGAG
**MosDEL**		
left homology region	ggggacaactttgtatagaaaagttggcCGAACCATCTGAGTACGTCG	ggggactgcttttttgtacaaacttgcGCTCAAAAATGTGTTGCTTCTGG
right homology region	ggggacagctttcttgtacaaagtggcgCTTGCAGAATTTTGATGGAAAC	ggggacaactttgtataataaagttgcGAAGCTTTCCTACAAAGAGC
xe3 5' end insertion	CGTCCTCACTTTCTGAGCTG	CCAATTCATCCCGGTTTCTG
xe3 3' end insertion	CCAATTACTCTTCAACATCC	CGACATTATTGATGTAACACC
**Probes for Nothern blotting**		
U1	GCACGCAGCCCCGATACGCA	
U2	CGATAAGAACAGATACTACAC	
U4	CGCACCTCGGCAAAGCCTCA	
U5	GGTTAAATGCAGAGGAACCAGAGT	

U6	ATTTGCGTGTCATCCTTGCGCAGG	
tRNA(gly)	GCTTGGAAGGCATCCATGCTGACCATT	
**3' RACE**		
RT primer	GACCGAGTGTAGCAAGCGAGGACTCGAGCTCAAGCCAAGCAGAAGACGGCATACGA	
U6 amplification	GACCGAGTGTAGCAAGCG	GTTCTTCCGAGAACATATAC
